# Trigeminal Neuralgia Due to Unilateral Early Bifurcated Superior Cerebellar Artery: Case Report and Literature Review

**DOI:** 10.7759/cureus.46201

**Published:** 2023-09-29

**Authors:** Joseph Silva, Amanda Viguini Tolentino Correa, Igor Alves da Silva, Camila Silva Pinto de Carvalho, Ricardo Ramina

**Affiliations:** 1 Neurosurgery, Instituto de Neurologia de Curitiba, Curitiba, BRA; 2 Neurologia, Faculdade Brasileira Multivix, Vitoria, BRA

**Keywords:** conservative treatment, case report, anatomical variant, superior cerebellar artery, trigeminal neuralgia

## Abstract

Trigeminal neuralgia (TN) is considered a debilitating pain syndrome resulting from a neurovascular conflict in the prepontine cistern, usually through compression of the trigeminal nerve by the superior cerebellar artery (SCA), resulting in neural pathology at the root entry zone. This is a case report of a patient whose TN symptoms were attributed to an anatomical variant of the SCA, managed successfully through conservative treatment. Anatomical variants of the SCA have been related to TN. However, this is the first reported case in the PubMed literature of primary TN due to an unilateral early bifurcated SCA treated conservatively with first-line sodium channel blockers with a good outcome.

## Introduction

The third edition of the International Classification of Headache Disorders (ICHD-3) defines trigeminal neuralgia (TN) as “a disorder characterized by recurrent unilateral brief electric shock-like pains, abrupt in onset and termination, limited to the distribution of one or more divisions of the trigeminal nerve, and triggered by innocuous stimuli” [1].

The incidence of TN is variable in the literature, affecting 12.6 to 27.0 per 100,000 person-years. It affects mostly women, with an average age of onset of 53 to 57 years old [2]. Converging evidence supports primary TN’s pathophysiological basis as a result of a neurovascular conflict in the prepontine cistern, with a compression of the trigeminal nerve by a blood vessel resulting in neural pathology at the root entry zone [2-4].

The superior cerebellar artery (SCA) is often considered the offending vessel related to TN symptoms [5]. It usually arises from the upper basilar artery as a single branch that bilaterally runs downward near the trigeminal nerve and travels posteriorly to supply the superior surface of the cerebellar hemispheres and superior vermis, the anterior medullary vellum, and part of the pineal gland, the tela chorioidea, and the dentate nucleus [5-8].

TN is initially managed conservatively but may also undergo neurosurgical procedures, such as microvascular decompression, in some cases. Carbamazepine and oxcarbazepine are first-choice medications for long-term pharmacological treatment with effective results [2].

This is a case report of a patient diagnosed with primary TN due to an anatomical variant of the SCA. To the best of our knowledge, this is the first reported case in the literature of a unilateral early bifurcated SCA related to TN symptoms treated conservatively.

## Case presentation

We present a case of a 42-year-old female patient with multiple episodes of short-lasting, electric shock-like facial pain at the distribution of the right mandibular division of the cranial nerve V that chronically progressed over two months. The paroxysmal pain attacks did not respond well to pain relievers such as metamizole and acetaminophen in regular dosages and were mostly triggered by chewing and brushing her teeth. Her medical history consisted of lumbar (L4-L5) intervertebral disc herniation in 2018. At initial presentation, both physical and neurological examinations were normal.

**Figure 1:**
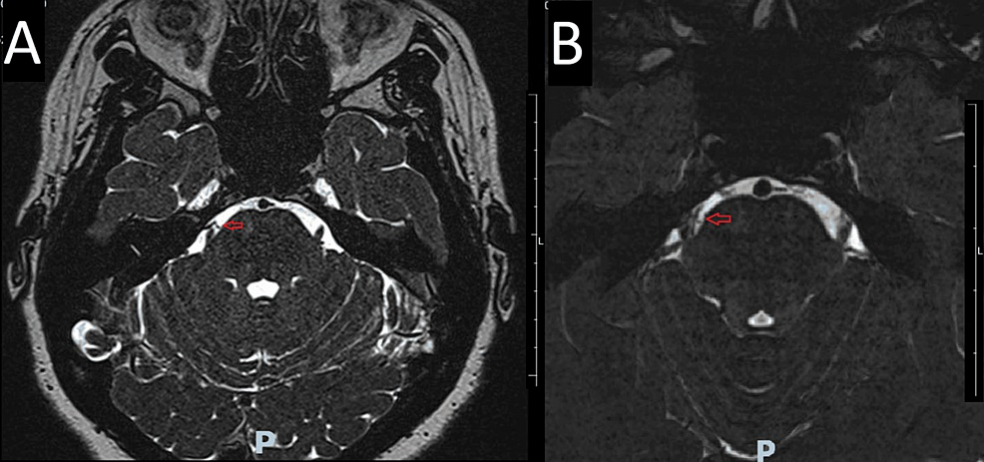
Axial magnetic resonance imaging (MRI) Axial view of an MRI T2 weighted image showing vascular conflict between the right trigeminal nerve and superior cerebelar artery in the entry zone (red arrows 1A and 1B).

**Figure 2:**
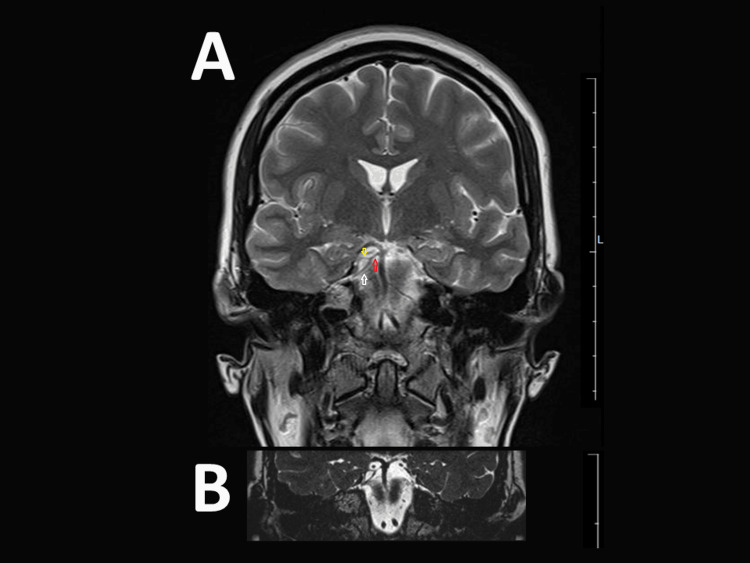
Coronal magnetic resonance imaging (MRI) Coronal view of an MRI T2 weighted image showing bifurcation of the right superior cerebelar artery (2A and 2B): Red arrow: superior cerebellar artery before bifurcation; Yellow arrow: superior branch of superior cerebellar artery; white arrow: inferior branch of superior cerebellar artery.

The patient underwent magnetic resonance imaging (MRI) of the brain, which revealed an early bifurcation of the right SCA (Figures 1, 2) in neurovascular contact with the ipsilateral trigeminal nerve. The left SCA presented no abnormalities. She was diagnosed with primary TN, and conservative treatment was initially proposed with 200 milligrams of carbamazepine three times a day. As her response to the treatment was satisfactory, there was no indication for any surgical approach. She currently remains pain-free at follow-up medical appointments.

## Discussion

The SCA is one of the major arteries of the posterior brain circulation [6]. Due to their close relation to cranial nerves III, IV, and V, the SCA morphologies can give rise to a wide clinical spectrum of neurovascular compression syndromes, such as trigeminal neuralgia and oculomotor nerve palsy, which is the first topic of this article [8]. As such, understanding de SCA anatomy is essential to the comprehension of the clinical phenomena caused by its variants [9].

Usually, the SCA is a single branch that rises between 18.5 and 27.2 mm from the origin of the basilar artery and goes in the direction of the cerebellum in order to give blood supply to the vermian and hemispheric cerebelar areas [8]. During its course, the SCA can be divided into four main segments: S1, anterior pontomesencephalic; S2, lateral pontomesencephalic; S3, cerebellomesencephalic; and S4, cortical segment. The closest relation to the CN V happens in the S3 segment [8].

The SCA happens to be a highly variable artery. Beyond its origin from the basilar artery, the SCA can also originate from the posterior cerebral artery (3% of cases) and from the internal carotid artery (less than 1% of people) [9].

Its morphology can also vary in interindividual comparison; it can be duplicated in 3%-30% of cases, making unilateral duplication more common. Bifurcation can also happen in up to 10% of people [8,9].

The contact between the SCA and V cranial nerve can happen in 45%-52% of cases, even though in up to 60% of cases, it does not produce symptoms of trigeminal neuralgia. It is postulated that the neurovascular compression could increase demyelination processes, leading to trigeminal neuralgia in some cases [8,9].

TN is considered a debilitating pain syndrome, often described as a burden for patients with a negative impact on the individual’s quality of life [2,9]. The neurovascular conflict in primary TN is mostly caused by the SCA (66.5% to 88%) [7] and has been related to anatomical variants of the SCA [10,11]. However, the authors present an unusual case of a patient with TN symptoms due to an anatomical divergence of the SCA conducted exclusively by the conservative approach.

Anatomical variants of the cerebellar arteries were described by Pekcevik et al. [11]. It is important to clarify that in our case, the offending SCA was not duplicated but instead presented an early bifurcation for the rostral and caudal branches, the last being mainly responsible for the vascular conflict.

Maarbjerg et al. propose a work-up and treatment algorithm for TN [3]. According to the authors, all TN patients must undergo an MRI of the brain and brainstem to consider a differential diagnosis, a laboratory test of renal and liver function and serum sodium levels, and an electrocardiogram to exclude atrioventricular block. [3]. First-line treatment with sodium channel blockers, such as carbamazepine and oxcarbazepine, should be considered. The conservative approach is effective in most TN cases. However, the side effects of the drugs, along with the need for high dosages to maintain pain relief, may be intolerable for some patients. The surgical approach, such as microvascular decompression, is then considered a reasonable next step [2-4]. The patient presented by the authors in this case report underwent an exclusive conservative treatment with an excellent outcome.

Literature reviews on anatomical variants of the SCA using radiological methods rather than post-mortem analysis are scarce [7,11]. Proper knowledge of the vascular anatomy of the vertebrobasilar system and its variations is important for surgical intervention of the cerebellopontine angle.

## Conclusions

TN is a debilitating pain syndrome caused by a neurovascular conflict in the prepontine cistern, often involving the SCA. Multiple anatomical variants of the cerebellar arteries were described in the literature, but few were related to TN symptoms. The authors report a rare case of a patient with an early bifurcation of the right SCA presenting with facial pain.
